# Alexithymia and Inflammatory Bowel Disease: A Systematic Review

**DOI:** 10.3389/fpsyg.2020.01763

**Published:** 2020-08-19

**Authors:** Gabriella Martino, Andrea Caputo, Peter Schwarz, Federica Bellone, Walter Fries, M. C. Quattropani, C. M. Vicario

**Affiliations:** ^1^Department of Clinical and Experimental Medicine, University of Messina, Messina, Italy; ^2^Department of Dynamic and Clinical Psychology, Sapienza University of Rome, Rome, Italy; ^3^Department of Medical Endocrinology, Copenhagen University Hospital, Copenhagen, Denmark; ^4^Department of Cognitive Sciences, Psychology, Education and Cultural Studies, University of Messina, Messina, Italy

**Keywords:** alexithymia, psychological distress, psychological functioning, adjustment, inflammatory bowel disease, chronic disease

## Abstract

**Background:** Given the role of alexithymia—as the inability to identify, differentiate, and express emotions—in chronic and immune-mediated illness, this systematic review analyzed the prevalence of alexithymia in patients with inflammatory bowel diseases (IBDs), mainly represented by Crohn's disease (CD) and ulcerative colitis (UC).

**Methods:** Preferred Reporting Items for Systematic Reviews and Meta-Analyses (PRISMA) guidelines were followed throughout this systematic review of the literature published between 2015 and 2020 in indexed sources from PubMed, PsycINFO, Scopus, and Web of Science databases. Search terms for eligible studies were: “Inflammatory bowel disease” AND “Alexithymia” [Titles, Abstract, Keywords]. Inclusion criteria were: articles written and published in English from 2015 and up to April 2020, reporting relevant and empirical data on alexithymia and IBD.

**Results:** The initial search identified 34 indexed scientific publications. After screening, we found that five publications met the established scientific inclusion criteria. Overall, the mean value of alexithymia ranged from 39 to 53.2 [Toronto Alexithymia Scale (TAS-20) score], thus mostly falling in non-clinical range for alexithymia (≤51). Comparisons of alexithymia between patients with UC and CD highlighted that patients with CD showed externally oriented thinking and difficulties identifying feelings to a greater extent. Regarding comparisons with other samples or pathologies, patients with IBD were more alexithymic than healthy controls and less alexithymic than patients with major depressive disorder, but no difference was found when compared with patients with irritable bowel syndrome (IBS). Then, regarding correlations with other variables, alexithymia was positively associated with anxiety and depression, as well as with psychopathological symptoms and somatic complaints.

**Conclusion:** This systematic review suggests that patients with IBD cannot be generally considered alexithymic at a clinically relevant extent. However, their greater alexithymic levels and its associations with psychological variables and somatic distress may suggest a reactivity hypothesis, in which living with IBD may progressively lead to impaired emotion recognition over time. Specifically, the relationship between IBD and IBS should be further explored, paying deeper attention to the clinical psychological functioning of CD, as IBD requires more emotional challenges to patients.

## Introduction

Increasing interest exists regarding the key role of psychological source and characteristics in protecting or exposing individuals to emotional distress. Clinical psychological features may affect the patient's ability to manage chronic diseases, leading to both lower compliance and adherence and predicting morbidity and mortality independently of several confounders (Caputo, 2013, 2019; Craparo et al., 2016; Conversano, 2019; Martino et al., 2019a,b; Merlo, 2019). On the other hand, medical conditions may impact mental health, leading to worse perceived quality of life which could in turn interfere with the ability to manage (Castelnuovo et al., 2015; Van Houtum et al., 2015; Di Giuseppe et al., 2018, 2019, 2020; Marchini et al., 2018; Catalano et al., 2019; Guicciardi et al., 2019; Rosa et al., 2019; Settineri et al., 2019; Lenzo et al., 2020; Martino et al., 2020a; Vicario et al., 2020). Among chronic illness, inflammatory bowel diseases (IBDs), mainly represented by Crohn's disease (CD) and ulcerative colitis (UC), show an increasing prevalence worldwide above all in Europe (CD, 322 per 100,000 persons; UC, 505 per 100,000 persons) and North America (CD, 319 per 100,000 persons; UC, 249 per 100,000 persons) (Molodecky et al., 2012; Jordan et al., 2016). IBD diagnosis generally occurs, without gender prevalence, debuts at age 10–40 years, showing frequently an unpredictable course. Particularly in CD, any part of intestines can be intermittently inflamed, while in UC, the inflammation is generally limited to the colon and rectum level only. This chronic medical condition leads to disabling symptoms such as fatigue, abdominal pain, diarrhea, and weight loss. The standard IBD treatment aims at pharmacological management of inflammation, with favor to an adequate compliance and adherence to regular medical controls and medications. In the most severe cases, in UC, the entire large bowel and rectum must be surgically removed, with typically subsequent transitory or permanent ileostomy, with relative psychological outcomes and worse perceived quality of life (Kiebles et al., 2010). Scientific data show that psychological features associated with specific lifestyle and environmental stressors impact both pathogenesis and relapses of IBD (Moreno-Jimènez et al., 2007; Boye et al., 2008). It is likewise reported that a high prevalence of alexithymia exists in patients suffering of chronic and immune-mediated illness characterized by somatic symptoms (Villoria et al., 2017; Erkic et al., 2018; Viganò et al., 2018), up to 35% in IBD, and that alexithymia is strictly related to clinical severity of gastrointestinal pathologies (Porcelli et al., 1995, 2014; Ferreiro et al., 2015). Alexithymia is a multidimensional construct, thought as the inability to differentiate between emotions, thoughts, and physiological replies to stimuli, which involves difficulties in recognizing and expressing emotions and externally oriented thinking (Nemiah and Sifneos, 1970; Sifneos, 1996; Taylor and Bagby, 2000; Tordeurs and Janne, 2000). Alexithymia is also considered as a personality trait which may appear in comorbidity with diverse physical and psychopathological disorders [Diagnostic and Statistical Manual of Mental Disorders, Fifth Edition (DSM-5) (Lumley et al., 2005, 2007; Mattila et al., 2009; Tolmunen et al., 2011; American Psychiatric Association, 2013; Brooks et al., 2019; Martino et al., 2019c, 2020b,c; Thavamani et al., 2019; Velotti et al., 2019; Orrù et al., 2020)], and it may assume the role of a temporary state linked to both psychopathological conditions and stress levels (Pollatos et al., 2011). It is also suggested that alexithymia is involved in the pathogenesis of numerous somatic disorders (Porcelli et al., 1996; Willemsen et al., 2008; Mazaheri et al., 2012; Marchi et al., 2019; Martino et al., 2019c), and it seems to be associated with both, depression and anxiety, in patients suffering from IBD (Graff et al., 2006; Filipović et al., 2007; Goodhand et al., 2012). Moreover, alexithymia and psychological distress as anxiety and depression may compromise the patient's compliance and adherence, leading to a severe clinical presentation and course of IBD (Sajadinejad et al., 2012a,b; Quattropani et al., 2019).

These evidences suggest patients living with alexithymia and IBD in comorbidity may experience significant relapses and worse course of IBD, which might be in part explained by difficulty in recognizing body signals, perceptions, and emotions (Mazaheri et al., 2012; Villoria et al., 2017; Viganò et al., 2018). Furthermore, the failure in recognizing emotion perceptions and physical symptoms could lead to a poor assessment and treatment of IBD, to an additional psychological and physical suffering, and to poor perceived quality of life, which in turn impair the patient's ability to cope and manage (Graff et al., 2006; Boye et al., 2008; Faust et al., 2012). Psychopathological comorbidity may be underestimated, and a deep, strategically oriented clinical psychological exploration is required to eventually highlight psychological features, such as alexithymic ones, considering the individual needs and outcomes of patients with IBD.

Hence, the purpose of this systematic review is to provide the current insights on the potential alexithymic characteristics of patients with IBD, underlining the clinical expressions of this complex. Particularly, our objective is to improve the awareness on the complex of alexithymia, IBD, and other related factors, supporting both psychologists and clinicians to carry out specific interventions to promote the adequate managing of IBD, encouraging psychological adjustment and well-being. A deeper understanding of this complex among patients may improve patients' knowledge of such chronic illness, way of feeling themselves, and perceived quality of life.

## Materials and Methods

### Data Source and Search Strategy

The review was executed according to the Preferred Reporting Items for Systematic Reviews and Meta-Analyses (PRISMA) (Liberati et al., 2009; Moher et al., 2009). In April 2020, PubMed, PsycINFO, Scopus, and Web of Science databases were explored, between 2015 and 2020, for eligible studies, in order to analyze the most recent literature, and the following terms were engaged: “Inflammatory bowel disease” AND “Alexithymia” [Titles, Abstract, Keywords].

### Publication Screening and Eligibility Criteria

After leading the first selection of the search, we eliminated study duplicates. During the second selection, all titles and abstracts were screened, and potential pertinent studies were identified for full-text review by two independent researchers in clinical psychology, for eligibility. Inclusion criteria were: scientific publications in English and with peer review published from 2015 and up to April 2020 reporting relevant and empirical data on alexithymia and IBD.

### Analysis Reviewed Publications and Data Synthesis

Methods were performed accordingly to the PRISMA guidelines (Liberati et al., 2009; Moher et al., 2009), seeing that the heterogeneity of the examined studies did not allow researchers to explore them by a meta-analysis. Researchers in clinical psychology independently reviewed the selected articles to confirm the reliability of the performed method. In order to provide a qualitative synthesis, carefully chosen studies were considered by matching substantial data and identifying the significant indexes of the measured variables.

## Results

### Search Result

**Figure 1:**
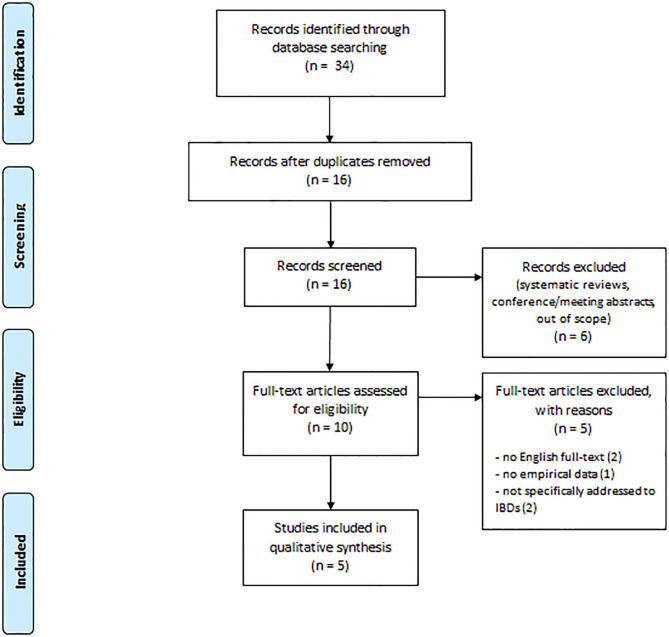
Preferred reporting items for systematic reviews and meta-analyses (PRISMA) flow diagram.

Figure 1 shows our search result and screening results according to PRISMA. Our search identified 34 publications. Eighteen publications were duplicates leaving our search with 16 publications for title and abstract review. After this review process, we identified in total 10 papers for full review. The excluded publications did not fulfill the inclusion criteria as two were systematic reviews, three were conference/meeting abstracts, and one was out of scope. Thus, the remaining 10 publications were full text reviewed, and five of them were removed for the following reasons: two did not provide English full text as they were written in Portuguese (Amorim and Guerra, 2018) and Russian (Mnatsakanyan et al., 2016), one did not provide empirical data about alexithymia (Edman et al., 2017), and two were not specifically addressed to patients with IBD (Cozzolongo et al., 2015; Schauer et al., 2019). We concluded that five papers could be included in our systemic review based on the inclusion criteria.

### Study Characteristics

We found that two out of five selected studies used a cross-sectional research design. Two studies specified that IBD diagnosis was based on the classical clinical, radiological, endoscopic, and histological criteria proposed by the European Crohn's and Colitis Organization (ECCO) (La Barbera et al., 2017; Yanartaş et al., 2019). All the studies reported specific inclusion/exclusion criteria for patient selection. The presence of previous psychiatric disorders and comorbidity of physical disease (e.g., neurological or oncologic pathologies, diabetes, rheumatoid arthritis) were the exclusion criteria reported in all the studies, followed by intellectual disability or cognitive impairment (La Barbera et al., 2017; Viganò et al., 2018; Yanartaş et al., 2019). Two studies included only patients being considered in clinical remission based on disease activity. Specifically, Viganò et al. (2018) adopted Crohn's Disease Activity Index (CDAI) and Mayo score to measure disease activity for CD and UC, whereas Fournier et al. (2020) used Harvey–Bradshaw Index (HBI) and UC activity index (UCAI), respectively. Concerning sample size, the retrieved studies included a number of patients with IBD ranging from 10 (Huang et al., 2016) to 170 (Viganò et al., 2018), with an average of 75 patients (*SD* = 62). Overall, the patients were aged 40 years on average, except for the study by Huang et al. (2016) that was addressed to adolescents and young adults (mostly 17–19 years old). Gender was fairly well distributed in the considered studies, with a mean of 48% of male patients (average of 35.2–55.3% in the studies). Beyond sociodemographics, three studies provided information regarding alcohol and substance use (La Barbera et al., 2017; Viganò et al., 2018; Yanartaş et al., 2019), whereas two studies reported further clinical data about IBD and type of therapy (La Barbera et al., 2017; Viganò et al., 2018). The main characteristics of the studies are reported in Table 1.

**Table 1:** Characteristics of included studies (in chronological order).

**References**	**Country**	**Purpose**	**Number of patients with IBD (% males)**	**Age range or mean age (SD) where indicated; years**	**Presence of comparison groups and sample size**
Huang et al. (2016)	USA (California)	Determine whether neural processing of somatic pain stimuli differs in adolescents and young adults with IBD and irritable bowel syndrome, as compared to healthy controls, and evaluate alexithymia, anxiety, depression, and pain catastrophizing.	10 (50%)	17–19	Yes (10 patients with irritable bowel syndrome, 10 healthy controls)
La Barbera et al. (2017)	Italy	Investigate the association between IBD and psychological dimensions such as personality traits, defense mechanisms, and alexithymia.	100 (51%)	Males: 40.7 (17.3); Females: 40 (14.1)	Yes (66 healthy controls)
Viganò et al. (2018)	Italy	Evaluate a broad spectrum of psychopathological symptoms and alexithymia levels in a group of outpatients affected by IBD in clinical remission, comparing CD and UC, and investigating the relationship with clinical and socio-demographic variables.	170 (55.3%)	47.10 (12.03)	No
Yanartaş et al. (2019)	Turkey	Investigate the effects of somatic and related symptoms, alexithymia, hypochondriasis, anxiety and depression on patients with major depressive disorder, irritable bowel syndrome, and IBD.	54 (35.2%)	36.46 (10.48)	Yes (102 patients with major depressive disorder, 51 patients with irritable bowel syndrome, 67 healthy controls)
Fournier et al. (2020)	France	Investigate whether difficulties in interoceptive abilities and difficulties in awareness of feelings are associated with the presence of irritable bowel syndrome, UC or CD, while checking for anxiety, depression, parasympathetic (vagus nerve) activity and cortisol levels.	39 (46.2%)	UC: 40.9 (10.8); CD: 40.3 (11.2)	Yes (24 patients with irritable bowel syndrome, 26 healthy controls)

*SD, standard deviation; UC, ulcerative colitis; CD, Crohn's disease; IBD, inflammatory bowel disease*.

### The Prevalence of Alexithymia in Patients With Inflammatory Bowel Disease

All the studies provided descriptive statistics about the Toronto Alexithymia Scale (TAS-20) total score in patients with IBD. Overall, the mean value of alexithymia ranged from 39 (Fournier et al., 2020) to 53.2 (La Barbera et al., 2017), thus mostly falling in the normal range, indicating no alexithymia, which scores ≤51 points. In this regard, it should be acknowledged that the highest alexithymia prevalence reported by La Barbera et al. (2017) was found in clusters of patients characterized by high neuroticism, impulsivity, and severe physical conditions. No study reported the percentage of alexithymic patients among the participants affected by IBD (with a cutoff score ≥61). Only two studies reported scores on TAS-20 dimensions (Viganò et al., 2018; Fournier et al., 2020). For each study, we calculated effect sizes (ESs) (expressed as the mean divided by the standard deviation of the sample) about the TAS-20 dimensions [Difficulty Identifying Feelings (DIF), Difficulty Describing Feelings (DDF), Externally Oriented Thinking (EOT)] as to compare the mean values on the different subscales as standardized scores. On average, patients with IBD scored higher on EOT (ES = 3.54) compared to both DDF (ES = 2.96) and DIF (ES = 2.79), with the latter having the lowest mean values.

### Comparisons of Alexithymia Between Patients With Ulcerative Colitis and Crohn's Disease

Two studies compared alexithymia levels among IBD subsamples. The study by Viganò et al. (2018) found no statistically significant difference on TAS-20 total score between patients with UC and CD, *d* = 0.13, 95% CI [−0.18, 0.43]; however, patients with CD generally reported higher values in EOT compared to their counterparts, *d* = 0.38, 95% CI [0.07, 0.69]. Also, the study by Fournier et al. (2020) found no difference on TAS-20 total score, *d* = 0.19, 95% CI [−0.44, 0.82], but DIF values were statistically significantly higher in patients with CD, *d* = 0.80, 95% CI [0.14, 1.45]. Besides, additional logistic regressions showed that none of the TAS-20 dimensions succeeded to explain for the presence of UC, whereas DDF was a significant predictor of CD [*W*_(1)_ = 6.16, *p* < 0.001], controlling for anxiety, depression, parasympathetic activity, and cortisol levels.

### Comparisons of Alexithymia Between Patients With Inflammatory Bowel Disease and Other Samples

Besides, four studies compared alexithymia of patients with IBD with other samples, such as healthy controls (Huang et al., 2016; La Barbera et al., 2017; Yanartaş et al., 2019; Fournier et al., 2020), patients with irritable bowel syndrome (IBS) (Huang et al., 2016; Yanartaş et al., 2019; Fournier et al., 2020), and patients with major depressive disorder (Yanartaş et al., 2019).

Regarding comparisons with healthy controls, in the study by Huang et al. (2016), patients with IBD had overall higher values of alexithymia than healthy controls with a very large effect size, *d* = 1.84, 95% CI [0.80, 2.89]. This difference is confirmed by the study by Yanartaş et al. (2019), despite with a smaller effect size, *d* = 0.48, 95% CI [0.12, 0.84]. Based on the findings by Fournier et al. (2020), it seems to be higher in patients with CD, *d* = 1.05, 95% CI [0.44, 1.67], than in patients with UC, *d* = 0.63, 95% CI [0.02, 1.25]. Besides, large-sized effects emerged in TAS-20 dimensions, indicating that patients with CD had higher DIF, *d* = 1.05, 95% CI [0.44, 1.67], and DDF values than healthy participants, *d* = 0.92, 95% CI [0.32, 1.53]. Instead, La Barbera et al. (2017) concluded that differences between the TAS-20 total score for patients and control participants were quite small and insignificant.

Concerning comparisons with patients with IBS, no difference on TAS-20 total score emerged with patients with IBD in any study (Huang et al., 2016; Yanartaş et al., 2019; Fournier et al., 2020), but patients with IBD were found to score lower on TAS-20 compared to patients with major depressive disorder, *d* = −1.19, 95% CI [−1.55, −0.84] (Yanartaş et al., 2019).

### Associations Between Alexithymia and Other Variables in Patients With Inflammatory Bowel Disease

Only two studies specifically examined the relationship between alexithymia and other measures in patients with IBD (Viganò et al., 2018; Yanartaş et al., 2019). In more detail, levels of anxiety and depression were evaluated in association with alexithymia. The study by Viganò et al. (2018) used the Hospital Anxiety and Depression Scale (HADS) and found a moderate association with anxiety (*r* = 0.52) and depression (*r* = 0.56). Whereas, Yanartaş et al. (2019) used the Beck Anxiety Inventory (BAI) and the Beck Depression Inventory (BDI) and confirmed small- tomedium-sized associations with anxiety (*r* = 0.52) and depression (*r* = 0.39), respectively. Besides this, psychopathological symptoms were assessed. Statistically significant, albeit small associations were detected between alexithymia and somatization (*r* = 0.23), obsessive–compulsive symptoms (*r* = 0.36), and global severity (*r* = 0.26) measured through the Symptom Checklist-90-Revised (SCL-90-R) (Viganò et al., 2018). As well, associations with hypochondriac worries and beliefs (*r* = 0.34) assessed through WI-7 (Whiteley Index-7) were found by Yanartaş et al. (2019). Then, the relationship between alexithymia and somatic symptoms was assessed, revealing statistically significant correlations with somatosensory amplification [*r* = 0.31; Somatosensory Amplification Scale (SSAS)] and functional somatic complaints [*r* = 0.46; Bradford Somatic Inventory-44 (BSI-44)]. Then, other small-sized statistically significant positive associations were found between alexithymia and clinical information, such as diagnostic delay (*r* = 0.21), utilization of IBD-specific poly-therapies (*r* = 0.20), and IBD extension (*r* = 0.16) (Viganò et al., 2018).

## Discussion

Our study aim was to evaluate the current evidence of alexithymia in patients with IBD. A limited number of scientific publications are focusing emotional capacities among patients with IBD, despite the well-acknowledged emotional issues and worse quality of life in persons living with chronic, idiopathic, inflammatory conditions compared to healthy population (Mählmann et al., 2017; Scott et al., 2020).

With regard to the prevalence of alexithymia, our findings suggest no evidence about a clinically meaningful impairment of emotional capacities in patients with IBD. However, it should be noted that only five studies addressing the review question were examined, and that none of them reported the percentage of alexithymic patients based on the widely used cutoff score ≥61. The standardized scores of TAS-20 dimensions across studies indicated externally oriented thinking as showing greater values, as a common trait that has been found in other physical diseases (Marty and De M'Uzan, 1963), which reflects a greater tendency to operative thinking and solving internal conflicts by external projection through actions (Perry et al., 2015; Porcerelli et al., 2017).

When comparing patients with UC and CD, no difference was found in the TAS-20 total score; however, patients with CD showed externally oriented thinking and difficulties identifying feelings to a greater extent. Besides, difficulty describing feelings was found to be a significant predictor of CD condition, even controlling for other potential confounders. Despite being preliminary, these findings seem to indicate some differences between IBD subpopulations. As suggested by Fournier et al. (2020), this could depend on the diverse psychophysiological functioning of UC and CD, as the digestive expression of the disease is less restricted and affects the entire gastrointestinal tract in CD. As a result, some patients whose CD is present closer to the stomach, may be more likely to experience disturbing symptoms, such as nausea and vomiting. Our result can be also explained in the light of anxiety and depression issues that are more frequently reported in patients with CD compared to their counterparts (Neuendorf et al., 2016), and of the greater flexibility of coping strategies in UC, which might be more adaptive for improving their psychological health (Sarid et al., 2018).

Compared with healthy samples, the present review highlights that patients with IBD have overall higher alexithymic levels, despite to a different extent in terms of effect size (Huang et al., 2016; Yanartaş et al., 2019; Fournier et al., 2020). This seems consistent with previous systematic reviews reporting issues of body image dissatisfaction, poor quality of life (Beese et al., 2019), and greater prevalence of anxiety and depression in IBD populations (Hyphantis et al., 2010; Neuendorf et al., 2016; Choi et al., 2019). This difference seems particularly relevant in patients with CD (Fournier et al., 2020), especially regarding difficulties identifying and describing feelings, thus supporting the previously discussed comparison between IBD subpopulations.

Instead, concerning comparisons with other clinical samples, our results provide further interesting insights. Overall, patients with IBD were found to be substantially similar to patients with IBS in terms of alexithymia (Huang et al., 2016; Yanartaş et al., 2019; Fournier et al., 2020). In this regard, Spiller and Major (2016) have proposed to consider IBD and IBS on the same spectrum rather than as separate entities because they share many common symptoms (e.g., abdominal pain and changed bowel habits) and have overlapping mechanisms of disease, such as increased gut permeability, increased production of mucosal mediators, and abnormal enteric nerves. Besides, patients with IBS and IBD show little differences in psychological distress or psychological risk factors if symptom activity is taken into account (Berens et al., 2019). Then, albeit some studies claiming more severe comorbid depressive and anxiety symptoms in IBS than in IBD (Geng et al., 2018), the bidirectional relationship of anxiety and depression or other altered psychological states in such populations has been scarcely explored (Rani et al., 2016).

In addition to this, the study by Yanartaş et al. (2019) showed lower alexithymic levels in patients with IBD compared to those with major depressive disorder. This result is not surprising given that depression and alexithymia are often described as similar constructs (Parker et al., 1991; Hemming et al., 2019), and there is a strong association between alexithymia and depression also in the general population (Honkalampi et al., 2000; Li et al., 2015). Besides this, most of literature supports the vulnerability hypothesis suggesting that alexithymia predisposes people to depression, rather than being reactive to depression (Hemming et al., 2019), whereas IBD represents an organic disease in which comorbid psychiatric issues are often secondary to disease itself (Pace et al., 2003).

About the associations between alexithymia and other variables, the current review highlights that alexithymia positively correlates with anxiety and depression in patients with IBD. Considering the high prevalence of anxiety and depressive symptoms in IBD populations, respectively equal to 35 and 22% (Neuendorf et al., 2016), this suggests to take into account alexithymia for planning and delivering psychological interventions. As well, some associations are detected between alexithymia and both psychopathological symptoms and somatic complaints, thus confirming the potential preventive role of emotion awareness and management for coping with illness. Then, other associations were found with IBD-related clinical data (e.g., diagnostic delay, IBD-specific therapy, and IBD extension), but they were overall small-sized and reported only in a single study (Viganò et al., 2018), thus requiring further investigation.

The current review has some inherent limitations that should be acknowledged. Among these, there is heterogeneity about the used inclusion/exclusion criteria, other study measures, and conducted analyses, as well as the size and characteristics of the samples (e.g., patients with active disease or in clinical remission). Another limitation is represented by the limited number of retrieved studies, which is also a strength of the study, since it highlights the lack of empirical findings and the need for further research in the field. Besides, as all the publications proposed cross-sectional research designs and relied on convenience samples, no generalization or inference can be made about the causal relationship between alexithymia and IBD, which would rather require longitudinal or experimental studies. However, the aim of this review was not assessing the impact of alexithymia in the development of such a clinical condition but providing room for discussion about its potential relevance in patients' psychological status and disease management.

## Conclusion

In conclusion, this systematic review suggests that patients with IBD cannot be considered as alexithymic to a clinically significant extent. In this regard, they are found to be quite similar to patients with IBS and less impaired in terms of emotion capacities than patients with major depressive disorder. However, empirical evidence emerges about their greater alexithymic levels compared to healthy participants and the associations found between such levels and other relevant psychological variables (i.e., anxiety, depression, somatization, obsessive–compulsive, and hypochondriac symptoms) and somatic distress. This overview may suggest a reactivity hypothesis according to which living with IBD may progressively lead to impaired emotion recognition and poor symbolization function about somatic experience over time. As alexithymia is a subjective condition that can be described along a continuum, its role thus remains meaningful. Future studies are needed to provide more robust empirical evidence on the issue. Specifically, the relationship between IBD and IBS should be further explored, and more attention should be paid to CD as such IBD condition seems to pose more emotional challenges to patients. In this regard, clinical psychological intervention is needed to enhance adjustment capacities in patients with CD as to promote quality of life and better adherence.

## Author Contributions

GM made significant contribution to the conception and design of the systematic review, to the acquisition, qualitative analysis, and synthesis of data by drafting both the first and revised versions of the manuscript. AC contributed to the qualitative analysis and synthesis of data by drafting both the first and revised versions of the manuscript. PS, FB, and WF gave significant contribution to draft part of the manuscript. MQ and CV revised the manuscript for intellectual content and gave the final approval of the manuscript to be submitted. All authors contributed to the article and approved the submitted version.

## Conflict of Interest

The authors declare that the research was conducted in the absence of any commercial or financial relationships that could be construed as a potential conflict of interest.
